# Exploring the Genetic Diversity and Population Structure of Wheat Landrace Population Conserved at ICARDA Genebank

**DOI:** 10.3389/fgene.2022.900572

**Published:** 2022-06-15

**Authors:** Muhammad Massub Tehseen, Fatma Aykut Tonk, Muzaffer Tosun, Deniz Istipliler, Ahmed Amri, Carolina P. Sansaloni, Ezgi Kurtulus, Muhammad Salman Mubarik, Kumarse Nazari

**Affiliations:** ^1^ Department of Field Crops, Ege University, Bornova, Turkey; ^2^ ICARDA-PreBreeding and Genebank Operations, Biodiversity and Crop Improvement Program, Rabat, Morocco; ^3^ Genetic Resources Program, CIMMYT, Texcoco, Mexico; ^4^ Turkey-ICARDA Regional Cereal Rust Research Center (RCRRC), Menemen, Izmir, Turkey; ^5^ Department of Biotechnology, University of Narowal, Narowal, Pakistan

**Keywords:** wheat landraces, genetic diversity, population structure, SNP markers, analysis of molecular variance (AMOVA)

## Abstract

Landraces are considered a valuable source of potential genetic diversity that could be used in the selection process in any plant breeding program. Here, we assembled a population of 600 bread wheat landraces collected from eight different countries, conserved at the ICARDA's genebank, and evaluated the genetic diversity and the population structure of the landraces using single nucleotide polymorphism (SNP) markers. A total of 11,830 high-quality SNPs distributed across the genomes A (40.5%), B (45.9%), and D (13.6%) were used for the final analysis. The population structure analysis was evaluated using the model-based method (STRUCTURE) and distance-based methods [discriminant analysis of principal components (DAPC) and principal component analysis (PCA)]. The STRUCTURE method grouped the landraces into two major clusters, with the landraces from Syria and Turkey forming two clusters with high proportions of admixture, whereas the DAPC and PCA analysis grouped the population into three subpopulations mostly according to the geographical information of the landraces, i.e., Syria, Iran, and Turkey with admixture. The analysis of molecular variance revealed that the majority of the variation was due to genetic differences within the populations as compared with between subpopulations, and it was the same for both the cluster-based and distance-based methods. Genetic distance analysis was also studied to estimate the differences between the landraces from different countries, and it was observed that the maximum genetic distance (0.389) was between the landraces from Spain and Palestine, whereas the minimum genetic distance (0.013) was observed between the landraces from Syria and Turkey. It was concluded from the study that the model-based methods (DAPC and PCA) could dissect the population structure more precisely when compared with the STRUCTURE method. The population structure and genetic diversity analysis of the bread wheat landraces presented here highlight the complex genetic architecture of the landraces native to the Fertile Crescent region. The results of this study provide useful information for the genetic improvement of hexaploid wheat and facilitate the use of landraces in wheat breeding programs.

## Introduction

Wheat crop is grown and cultivated on more land area than other commercial crops and provides basic human nutrition. Wheat as food is a source of energy and protein for about two billion people and plays an indispensable role in food security as it provides 20% of world caloric consumption (Lawlor and Mitchell, 2000; Bhatta et al., 2017). With the growing population, climate constraints, changes in lifestyles, globalization of taste, urbanization, and development, there is a need for genetic improvement in wheat for yield and quality. It has been estimated that there should be a 50% increase in wheat production by 2050 (Grassini et al., 2013; Ray et al., 2013; Marcussen et al., 2014; Yang et al., 2020). As the environment plays an important role in wheat performance, it is difficult to find an area free from (biotic/abiotic) stress (Fan et al., 2012). Therefore, it is essential to measure each potential line performance across many years and under wide geographical regions to get higher yield potential.

Hexaploid wheat is comprised of three genomes (A, B, and D) as a result of natural hybridization and thus does not share any direct wild ancestor with the same genomic constitution (Terasawa et al., 2009). *Triticum urartu*, *Aegilops speltoides*, and *Aegilops tauschii* are the parental sources of genomes A, B, and D, respectively (Kunert et al., 2007; van Ginkel and Ogbonnaya, 2007; Charmet, 2011; Nielsen et al., 2014). Hence, it is essential to protect and sustain the existing genetic variability among hexaploid wheat accessions.

The success of breeding programs relies on the presence of significant genetic variability in a source population. High genetic variability boosts the chances to select superior genotypes from a population (Khan et al., 2015). On the other hand, a narrow genetic base is one of the major constraints, as it makes the plants more vulnerable to stress (biotic/abiotic) conditions (Novoselović et al., 2016; El-Esawi et al., 2018; Tehseen et al., 2021b). Continuous breeding practices such as artificial selection for the quality and yield traits have narrowed the genetic diversity in bread wheat over the past years (Novoselović et al., 2016; El-Esawi et al., 2018). Collection and evaluation of a large number of landraces from different regions to dissect the genetic diversity and variability would be the first step to broadening the genetic base of the wheat crop. Landraces are locally adapted distinct species and produce relatively higher yields under natural conditions with low or no agricultural input and show maximum resistance to the stress environments (Zeven 1998). They are supposed to be the best source for transferring their economically important traits to elite cultivars of different crops such as maize, legumes, rice, and wheat (Hargrove and Cabanilla, 1979; Feldman and Sears, 1981; Isemura et al., 2001; Malvar et al., 2004; Reif et al., 2005; Zhang et al., 2009). Therefore, the characterization of the genetic diversity of landraces can provide precious information that can be utilized to broaden the narrow genetic base in crops (El-Esawi et al., 2018).

Morphological traits are not the best indicators to evaluate genetic diversity as largely influenced by environmental conditions (Yang et al., 2020). Hence, DNA molecular markers can now be used to tag and locate numerous interacting genes that regulate complex traits. Combining the marker-assisted selection (MAS) with conventional methods of plant breeding schemes can enhance the overall selection gain and therefore increase the efficiency of breeding programs. Various molecular markers have been used to find the genetic diversity among different wheat genotypes such as amplified length fragment polymorphism (AFLP) (Lage et al., 2003; Das et al., 2016; Bhatta et al., 2017), randomly amplified polymorphic DNA (RAPD) (Khan et al., 2015), inter-simple sequence repeats (ISSRs) (Khan et al., 2015), simple sequence repeat (SSR) (Belete et al., 2020), and single nucleotide polymorphisms (SNPs) (Bhatta et al., 2017; Joukhadar et al., 2017; Mourad et al., 2020). SNPs are one of the most common marker systems for evaluating genetic diversity which provide numerous polymorphisms in single plant genomes (Yang et al., 2020). This study was initiated and fulfilled to address subsequent objectives: 1) to decipher the population structure and unlock the genetic diversity among the bread wheat landraces from eight different countries, 2) to provide useful information about the genetic diversity and population structure of these landraces for future breeding programs, and 3) to evaluate the different approaches used for determining the population structure. The outcomes of this study will help in the utilization of these landraces effectively to broaden the genetic base of hexaploid wheat and facilitate the discovery of new genomic regions providing resistance to economically important biotic and/or abiotic stresses.

## Material and Methods

### Plant Material

A wheat diversity panel containing 600 landraces from the International Centre for Agricultural Research in the Dry Areas (ICARDA) was used in this study. The landraces in the panel were obtained from 8 countries, which were Syria (376), Turkey (157), Iran (47), Greece (7), Iraq (7), Spain (3), Jordan (2), and Palestine (1).

### DNA Isolation and Genotyping

Fresh leaves were collected from 10-day-old seedlings in labeled Eppendorf Tubes and sunk immediately into liquid nitrogen; then, they were transferred to the lab and stored at −80°C. Leaf samples were grounded using a tissue lyser (TissueLyser II from QIAGEN). Genomic DNA was extracted from 0.1 g powdered leaf samples by using the cetyltrimethylammonium bromide (CTAB) method (Doyle and Doyle, 1991). Extracted DNA was dissolved in 100 μl tris-EDTA (TE) buffer. The DNA samples were run on 1% agarose gel for purity test, and a spectrophotometer (NanoDrop ND-1000) was used to quantify the DNA. The samples were then stored at −80°C.

A high-throughput genotyping by sequencing (GBS) method using Diversity Arrays Technology (DArT) (Sansaloni et al., 2011) was used for all samples at the Genetic Analysis Service for Agriculture (SAGA) at the International Maize and Wheat Improvement Center (CIMMYT) in Mexico and supported by the CGIAR Research Program (Sansaloni et al., 2011).

### Population Structure Analysis

To reveal the population structure of the wheat diversity panel, a model-based Bayesian cluster analysis was performed with STRUCTURE software (v. 2.3.4) (Pritchard et al., 2000). The program was run for ten replicates for each putative subpopulation ranging from k = 1 to k = 10 under the admixture model of population structure and was assessed with a burn-in period of 50,000 followed by 50,000 Markov Chain Monte Carlo (MCMC) replications. The best K value was used to identify the optimum number of clusters/subpopulations. The best K value was estimated as Delta K (ΔK) from Structure Harvester (Evanno et al., 2005) using the log probability of the successive structure iterations. For the optimal K value, to generate both individual and population Q-matrices by using the membership coefficient matrices of three replicates from the STRUCTURE, the CLUMPP (Jakobsson and Rosenberg, 2007) was used. Afterward, the DISTRUCT program was used to generate the bar plot from the integrated geographical information (Jakobsson and Rosenberg, 2007).

The discriminant analysis of the principal components (DAPC) was used as a second approach to analyze the population structure. DAPC uses the K-means clustering of principal components to identify the group of each individual. The numbers and nature of the clusters are assessed using the Bayesian Information Criterion (BIC). The DAPC analysis was conducted by using the R package “adegenet” (Jombart et al., 2010) in R studio (R Development Core Team 3.0.1., 2013).

### Genetic Diversity and Analysis of Molecular Variance

Various diversity parameters like the number of different alleles (*Na*), number of effective alleles (*Ne*), Shannon’s index (*I*), diversity index (*He*), unbiased diversity index (*uHe*), and percentage of polymorphic loci (*PPL*) were measured using GenAIEx v. 6.503 (Peakall and Smouse, 2006) to analyze the genetic variation among the 600 bread wheat landrace from 8 countries. The subpopulations obtained from STRUCTURE and DAPC were used for analysis of molecular variance (AMOVA), the calculations of Nei’s genetic identity, and genetic distance among populations. R package “adegenet” was used to perform the principal component analysis (PCA), while “poppr” (Kamvar et al., 2014) was used to construct the minimum spanning network (MSN) and neighbor-joining phylogenetic tree based on simple matching dissimilarity coefficient without the assumption of an evolutionary hierarchy.

## Results

### Single Nucleotide Polymorphism Markers Distribution

**FIGURE 1:**
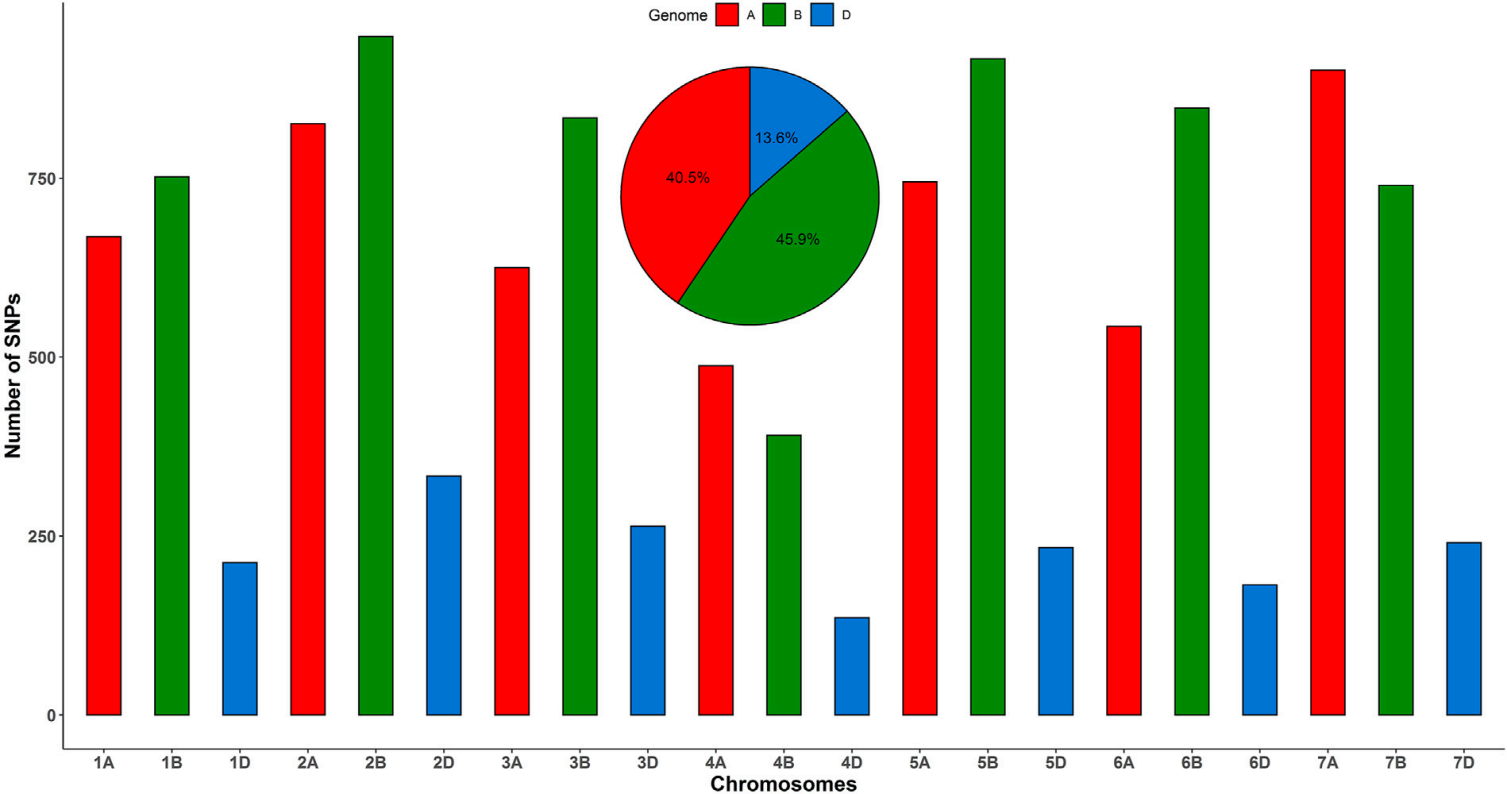
Distribution of 11,830 SNPs across 21 chromosomes of bread wheat landraces from 8 countries.

A total of 600 landraces collected from 8 countries were genotyped using the GBS method. A set of 25,169 SNPs were discovered. The SNPs were filtered for quality control (QC) based on >20% missing data and minor allele frequency (MAF) <5%. After QC and SNP filtering, the set of the 11,830 SNPs on the 21 chromosomes was selected for analysis. The highest number of markers was mapped on the B genome (5,430) followed by the A genome (4,796) and D genome (1,604) (Figure 1). The highest number of markers was found on chromosome 2B (948) followed by 5B (917) and 7A (901), while the lowest number of markers was mapped on 4D (136) followed by 6D (182) and 7D (241) (Figure 1).

### Population Stratification and Genetic Relationships

**FIGURE 2:**
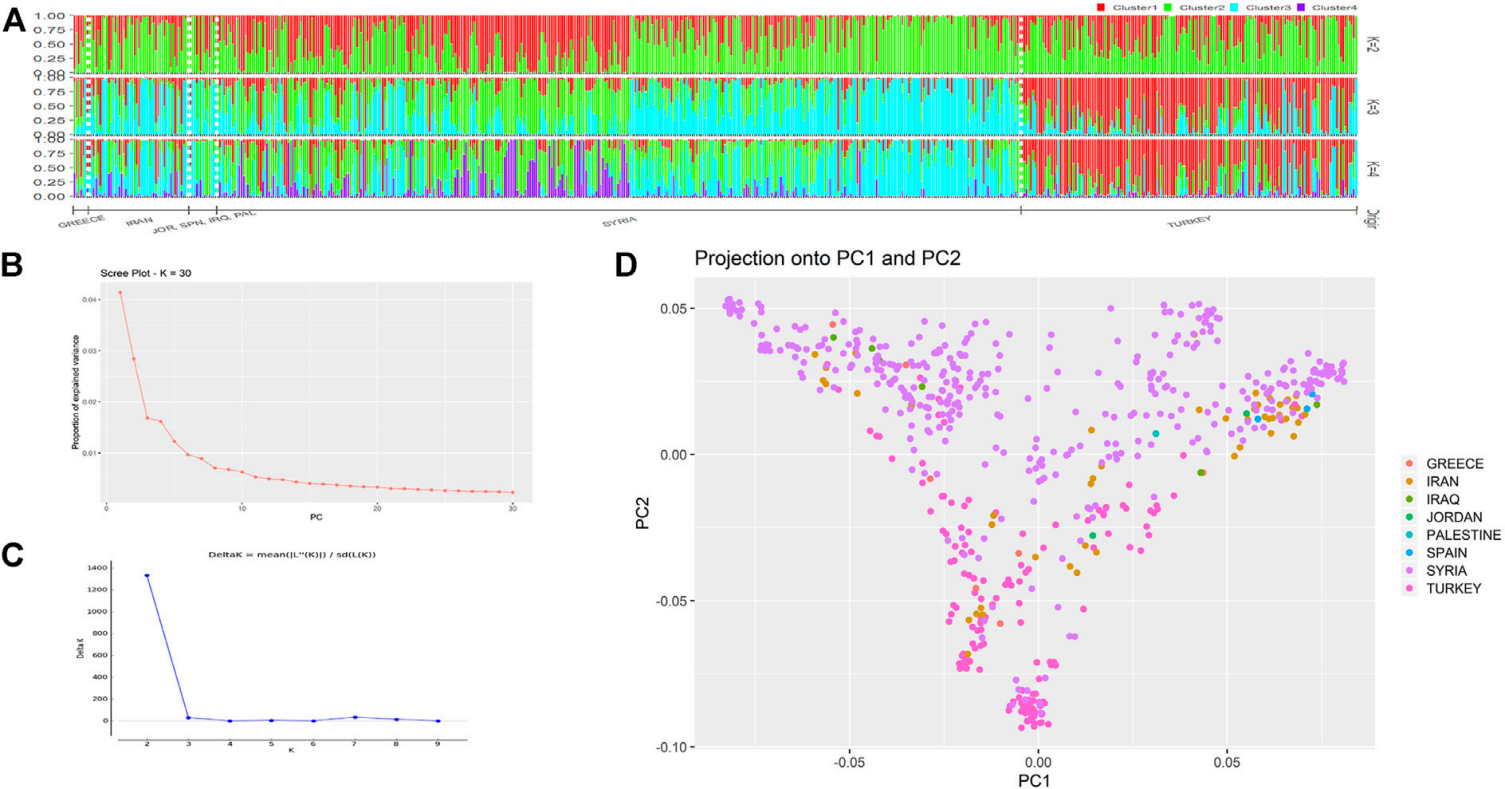
**(A)** Population structure of 600 bread wheat landraces for k = 2, 3, and 4. Different colors represent the subpopulations, and each bar represents the estimated membership of a single genotype. The horizontal line under the figure indicates the geographic origins of the landraces. **(B)** The scree plot of ΔK against the proportion of explained variance states the optimal subpopulation number in DAPC analysis. **(C)** The plot of K against ΔK to determine the optimum K value for STRUCTURE analysis. **(D)** The PCA of 600 bread wheat landraces.

Two different approaches, STRUCTURE and DAPC were used to identify the underlying stratification in the whole population panel. For the STRUCTURE program as the first approach, the optimum number of subpopulations was determined by the change of likelihood (ΔK). The results suggested that the optimum population structure was at K = 2. To find the optimal subpopulation number, the plot of K against ΔK (Figure 2C) was used. The plot showed that the optimal K value was 2, which was the peak of the graph. Among the two subpopulations, 362 landraces were grouped in subpopulation 1, while 238 landraces were grouped in subpopulation 2 (Table 1). It can be seen from STRUCTURE results that the landraces were not grouped based on their geographic origin (Figure 2A). For example, the landraces from Syria and Turkey were grouped in both subpopulation 1 and subpopulation 2. The values of fixation index (Fst) as the indicator of the genetic variation among the landraces in each cluster were 0.23 and 0.21 for subpopulation 1 and subpopulation 2, respectively.

**TABLE 1:** The STRUCTURE results of 600 bread wheat landraces for the fixation index (Fst), average distances (expected heterozygosity/He), gene flow (Nm), and the number of genotypes assigned to each subpopulation.

Population	Inferred clusters	Mean Fst	Exp. Het	Nm	No. of genotypes
Pop1	0.619	0.2307	0.31	0.833	362
Pop2	0.381	0.2078	0.3455	0.953	238

The results of the PCA revealed that the landraces were grouped into three groups. The first, second, and third PCs explained 15, 18, and 22% of the total variation, respectively.

**FIGURE 3:**
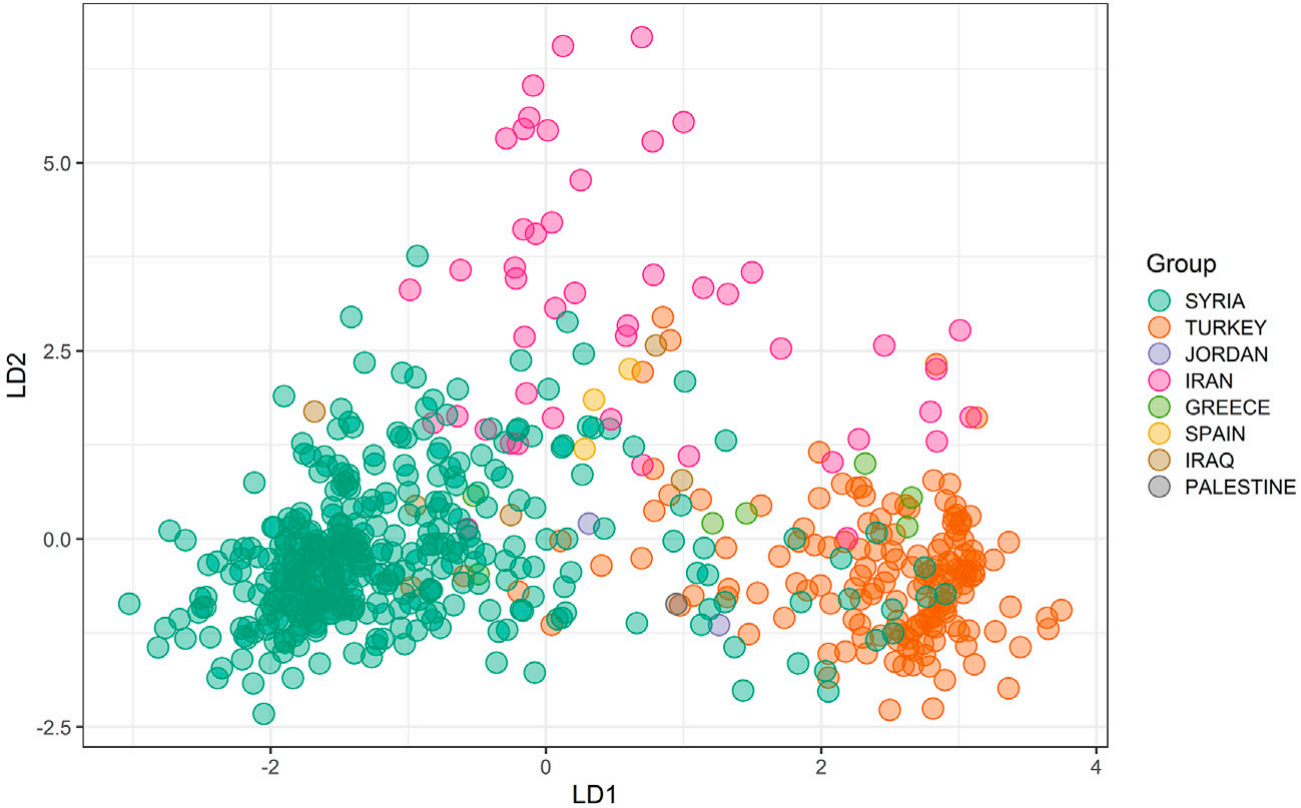
Inference of the subpopulations by DAPC analysis grouping landraces from different countries together.

The DAPC was used as the second approach, and the scree plot of ΔK against the proportion of explained variance showed that the landraces were divided into at least three subpopulations (Figure 2B). The three subpopulations comprised 181, 193, and 226 landraces, respectively. According to the DAPC analysis, the landraces from Syria, Turkey, and Iran were in distinct groups with mild admixture. The landraces from Greece were genetically more similar to Turkish landraces, whereas the Iraqi landraces were found to have similarities with both Iranian and Syrian landraces (Figure 3).

**FIGURE 4:**
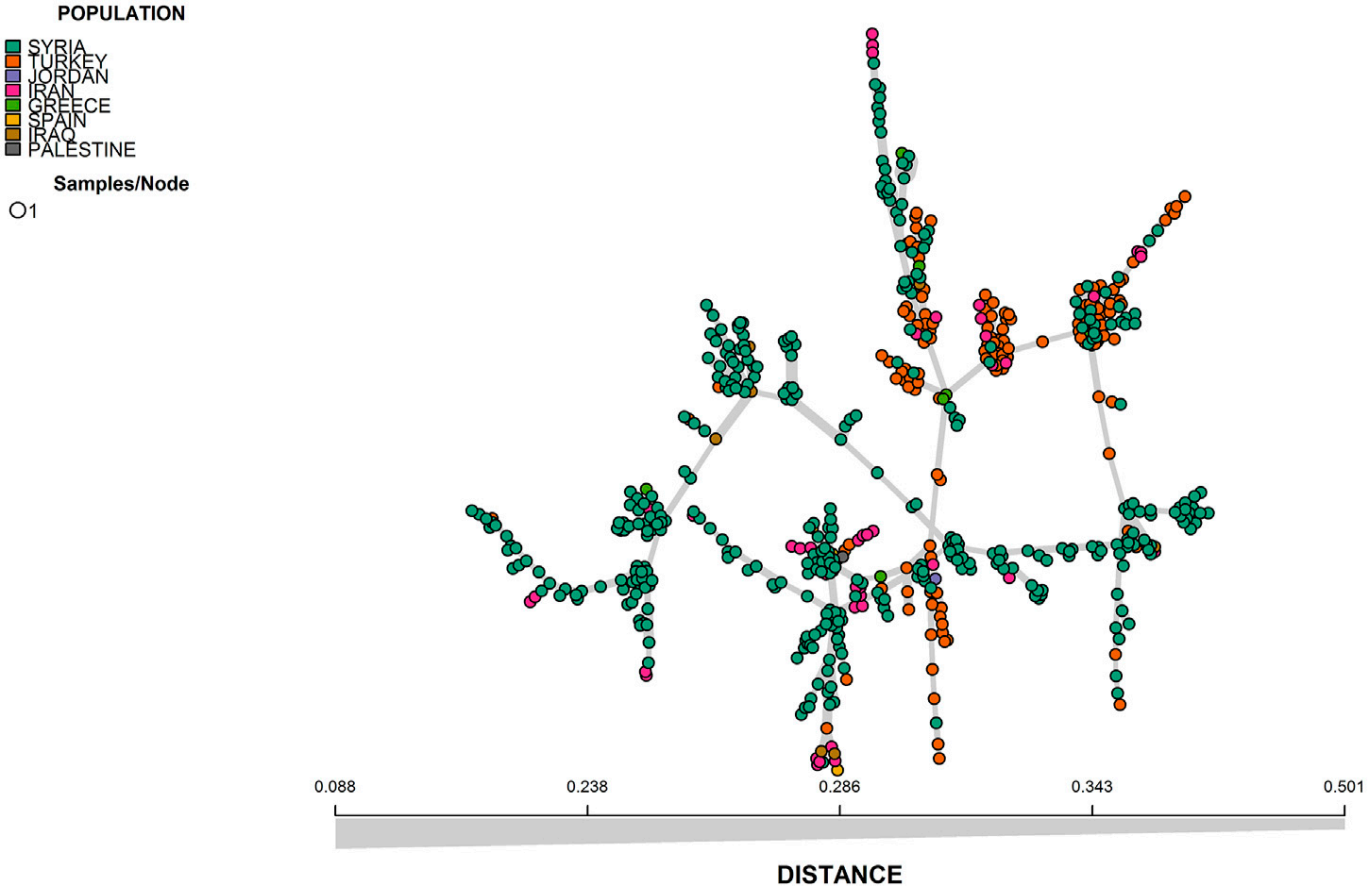
Minimum spanning network (MSN) of 600 bread wheat landraces.

**FIGURE 5:**
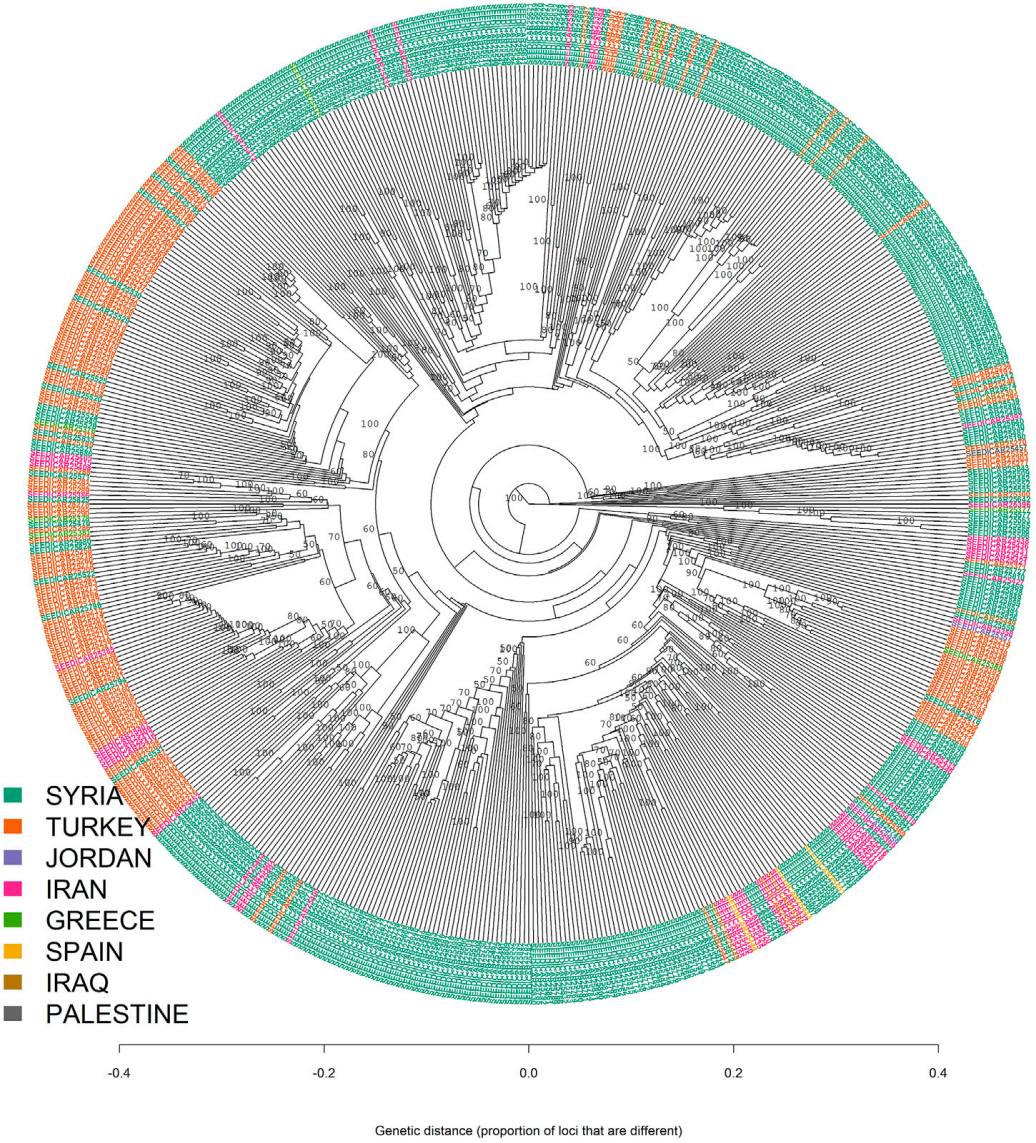
Neighbor-joining clustering of 600 bread wheat landraces.

The results from both STRUCTURE and DAPC analyses showed that there was an admixture between the different geographic regions which can be seen from the results of the minimum spanning network (MSN) and neighbor-joining based clustering analyses (Figures 4, 5).

### Genetic Differentiation Among Subpopulations

Three AMOVAs were generated based on the results of STRUCTURE and DAPC, as well as on the geographic origin of the landraces (Table 2.). The STRUCTURE-based AMOVA showed that a small amount of genetic variation (2.5%) was observed between the two subpopulations and a big portion of genetic variation (97.5%) was observed within the subpopulations. The genetic variance between two subpopulations was 4.2% in DAPC-based AMOVA, which implies 95.8% genetic variance within three subpopulations. The highest genetic variance among the subpopulations (5.9%) was obtained from origin-based AMOVA (Table 2).

**TABLE 2:** Analysis of molecular variance (AMOVA) revealing genetic diversity in bread wheat landraces.

Method	Source	df	SS	MS	Est. var	%
Model based (STRUCTURE)	Among Pops	1	26356.26	26356.26	80.95107	2.5
	Within Pops	598	1858696	3108.187	3108.187	97.4
	Total	599	1885052		3189.138	1
Distance based (Cluster, DAPC)	Among Pops	2	59508.97	29754.49	133.9296	4.2
	Within Pops	597	1825543	3057.861	3057.861	95.8
	Total	599	1885052		3191.791	1
Based on origin	Among Pops	7	147767.9	21109.71	328.5178	5.9
	Within Pops	592	3622336	6118.811	6118.811	94.1
	Total	599	3770104	6293.997	6447.329	1

df; degrees of freedom, SS; Sum of squares, MS; Mean square, Est. var; Estimated variance.

The low genetic variability among the subpopulations implies a high amount of gene flow between the landraces evaluated in this study. Besides, the higher values of genetic variability within the populations for STRUCTURE (97.5%), DAPC (95.8%), and origin-based AMOVA (95.5%) suggest that the landraces from eight countries shared common ancestries and were highly admixed (Table 2).

The genetic distances were calculated to decipher the levels of diversity between the subpopulations (Table 3). The genetic distance between the two subpopulations formed by STRUCTURE was 0.013, which implies a high admixture level. In terms of DAPC, the maximum genetic distance (0.032) was calculated between subpopulations 1 and 3, while the minimum genetic distance (0.015) was found between subpopulations 2 and 3 (Table 3). The clustering in terms of geographic origins yielded the maximum genetic distance of 0.289 between the landraces from Palestine and Spain followed by a genetic distance of 0.246 between landraces from Palestine and Jordan. On the other hand, the lowest genetic distance was observed between Syria and Turkey (0.013) (Table 3).

**TABLE 3:** Nei’s genetic identity (above diagonal) and genetic distance (below diagonal).

Model									
Model based (STRUCTURE)		**Pop1**	**Pop2**						
	**Pop1**		0.987						
	**Pop2**	0.013							
Distance based (Cluster, DAPC)		**Pop1**	**Pop2**	**Pop3**					
	**Pop1**		0.977	0.969					
	**Pop2**	0.023		0.985					
	**Pop3**	0.032	0.015						
Based on origin		**SYRIA**	**TURKEY**	**IRAN**	**GREECE**	**IRAQ**	**JORDAN**	**PALESTINE**	**SPAIN**
	**SYRIA**		0.987	0.948	0.915	0.908	0.870	0.818	0.889
	**TURKEY**	0.013		0.954	0.916	0.920	0.872	0.819	0.897
	**IRAN**	0.053	0.047		0.937	0.912	0.862	0.855	0.848
	**GREECE**	0.089	0.087	0.065		0.878	0.833	0.804	0.819
	**IRAQ**	0.096	0.083	0.092	0.130		0.841	0.803	0.847
	**JORDAN**	0.139	0.137	0.148	0.182	0.173		0.782	0.815
	**PALESTINE**	0.201	0.200	0.157	0.219	0.219	0.246		0.749
	**SPAIN**	0.118	0.109	0.165	0.199	0.165	0.205	0.289	

### Genetic Diversity Across Subpopulations

The mean values for the number of different alleles (*Na*) and number of effective alleles (*Ne*) of two subpopulations determined by STRUCTURE were 1.993 and 1.476, respectively (Table 4). The averages of the Shannon index (*I*), diversity index (*He*), and unbiased diversity index (*uHe*) of the two subpopulations were 0.443, 0.288, and 0.288, respectively. STRUCTURE-based analysis showed that subpopulation 2 (*I* = 0.445, *He* = 0.289, *uHe* = 0.290) had a slightly higher genetic diversity than subpopulation 1 (*I* = 0.441, *He* = 0.286, *uHe* = 0.286).

**TABLE 4:** Mean of different genetic parameters: number of different alleles (Na), number of effective alleles (Ne), Shannon’s index (I), diversity index (He), unbiased diversity index (uHe), and percentage of polymorphic loci (PPL) in each of the two subpopulations.

Method	Pop	Na	Ne	I	He	uHe	PPL (%)
Model based (STRUCTURE)	**Pop1**	1.988	1.472	0.441	0.286	0.286	99.38
	**Pop2**	1.997	1.479	0.445	0.289	0.290	99.84
	**Mean**	1.993	1.476	0.443	0.288	0.288	99.61
Distance based (Cluster, DAPC)							
	**Pop1**	1.970	1.434	0.410	0.264	0.266	98.35
	**Pop2**	1.987	1.483	0.449	0.292	0.293	99.32
	**Pop3**	1.992	1.474	0.440	0.286	0.287	99.49
	**Mean**	1.983	1.464	0.433	0.281	0.282	99.05
Based on origin							
	**SYRIA**	1.988	1.474	0.442	0.287	0.287	99.40
	**TURKEY**	1.987	1.472	0.440	0.285	0.287	99.22
	**IRAN**	1.762	1.412	0.381	0.248	0.254	85.63
	**GREECE**	1.242	1.320	0.282	0.188	0.209	54.02
	**IRAQ**	1.021	1.253	0.222	0.148	0.166	42.47
	**JORDAN**	0.614	1.137	0.117	0.080	0.107	19.39
	**PALESTINE**	0.297	1.000	0.000	0.000	0.000	0.00
	**SPAIN**	0.738	1.177	0.154	0.104	0.129	26.79
	**Mean**	1.887	1.447	0.416	0.270	0.273	53.36

For the DAPC approach, the mean *Na* value of the three subpopulations was 1.983 and the mean *Ne* was 1.464. The averages of *I*, *He*, and *uHe* were 0.433, 0.281, and 0.282, respectively (Table 4). According to the DAPC results, subpopulation 2 showed a higher diversity (*I* = 0.449, *He* = 0.292, *uHe* = 0.293) than subpopulations 1 and 3.

The mean values of the genetic indices obtained by geographic origin-based grouping for seven countries (Palestine was not taken into consideration since it has one landrace) were *Na* = 1.887, *Ne* = 1.447, *I* = 0.416, *He* = 0.270, and *uHe* = 0.273. The landraces originating from Syria showed the highest diversity with the diversity parameters of *I* = 0.442, *He* = 0.287, and *uHe* = 0.287, and the lowest genetic diversity was observed within Jordan landraces (*I* = 0.117, *He* = 0.080, and *uHe* = 0.107).

### Clustering *via* Geographic Origin

**FIGURE 6:**
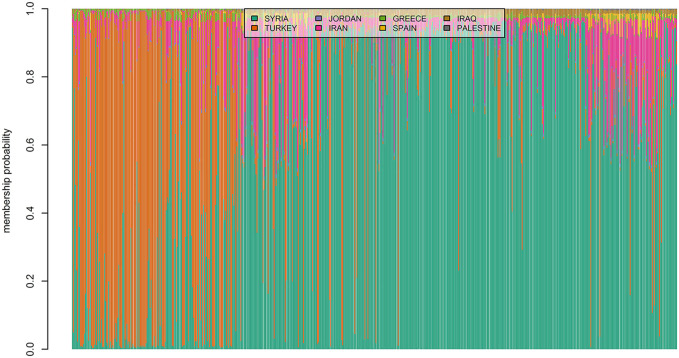
Estimated population membership probability of 600 bread wheat landraces from eight countries where each bar represents a landrace.

The membership coefficients of 600 landraces were presented as bar plots in Figure 6. The graph had two major groups and one minor group which suggested that almost all landraces had a similar ancestry to the genotypes from Syria or Turkey. Some of the landraces from Iran had genetic similarities with Turkey and Syria. For example, almost all genotypes from Iran were somewhat genetically similar to the landraces from Syria. However, a relatively low admixture level was observed for the landraces originating from Turkey. Also, it can be seen from Figure 6 that the majority of genotypes from Spain admixed with the landraces from Iran and Syria.

## Discussion

Global wheat production is facing new challenges in terms of climate change and biotic stress resistance; thus, the studies of genetic diversity could prove to be helpful for the effective conservation and improvement of the existing germplasm (Rao and Hodgkin, 2002). In breeding programs, the breeder’s emphasis is on mainly increasing and sustaining wheat production, and the enhanced breeding and conversation strategies can be used to broaden the genetic base of the wheat crop by the information derived from genetic diversity, population structure, and their relationships (El-Esawi et al., 2018). Wheat landraces are used in several wheat breeding programs, as they provide unique potential and diversity of key genes controlling both biotic and abiotic stresses (Manickavelu et al., 2016).

The current study was conducted on a total of 600 bread wheat landraces from 8 different countries preserved at ICARDA's genebank to evaluate the genetic diversity and population structure through GBS-derived SNPs. It could be beneficial to open up the genetic constituents to identify novel genes and loci to improve plant resistance and further breeding programs. A total of 11,830 SNP markers distributed across the hexaploid wheat genomes A, B, and D were used to evaluate the population structure of the wheat landraces. Greater sequence diversity was found in genome B (5,430 SNPs), followed by genome A (4,796 SNPs), and D (1,604 SNPs), and these findings are in agreement with previous studies (Poland et al., 2012; Alipour et al., 2017). The results showed that the D genome is the least polymorphic probably due to the low frequency of recombination rates (Chao et al., 2009; Alipour et al., 2017). The low polymorphism of markers on the D genome is unique to wheat than to its ancestor *Aegilops tauschii* (Akhunov et al., 2010). The numbers of SNPs in genomes B and A were more than three times higher than in genome D, which is similar to many previous reports (Iehisa et al., 2014; Eltaher et al., 2018; Bhatta et al., 2019). Similar to the previous study, the current study also reported the least number of SNPs on homologous chromosome 4 in all three genomes (Rimbert et al., 2018). After QC, the minimum number of SNPs was found on chromosome 4D, which is similar to previously reported studies (Alipour et al., 2017; Rimbert et al., 2018).

The understanding of the population structure is crucial for further downstream analysis, for example, genome-wide association studies (GWAS). The evaluation of genetic diversity also provides vital information which can help in the preservation strategies and broadening of the genetic base of crops (Eltaher et al., 2018; Tehseen et al., 2021a). The presence of subpopulations in the panel can be attributed to the selection of desirable traits and genetic drift (Kumar et al., 2020). In the current study, the population stratification estimated by STRUCTURE identified two potential subpopulations in the landraces panel. The two subpopulations were broadly divided into Syrian and Turkish landraces. Although a large number of landraces (n = 533) were collected from these two countries and two subpopulations seemed like an acceptable clustering, nevertheless, the two populations were highly admixed with no clear differentiation, therefore further analyses were conducted in order to find the genetic diversity and population clustering of these 600 landraces. Furthermore, it has been reported that the value of k = 2 in STRUCTURE sometimes means that the STRUCTURE could not correctly identify the genetic structure of the population (Janes et al., 2017). We used DAPC and PCA to further dissect the true structure of the landrace population. The PCA and DAPC results identified three potential subpopulations. Although there was admixture within the populations, the clusters were primarily based on landraces from Syria, Turkey, and Iran, which was initially expected from the population as well because the landraces were mainly collected from these three geographical regions, and the landrace native to these lands were supposed to show some overlapping and genetic differentiation as previously reported by Yang et al. (2020). Therefore, it was concluded that the results of DAPC and PCA were more precise in comparison with STRUCTURE. A previous study of 804 bread wheat accessions from 30 different countries identified that the European accessions were separated from the majority of Asian and Middle Eastern accessions and the latter showed overlapping (Winfield et al., 2018). Similarly, Balfourier et al. (2007) used 3,942 wheat genotypes originating from 73 countries, characterized them with a set of 38 SSR markers, and observed a close relationship between the accessions from Turkey, Iran, and Iraq. Another study of 78 wheat landraces from 22 countries reported that the landraces were primarily divided into Asian and European clusters; furthermore, the landraces from Turkey and Iran were placed in the same subgroup thus further confirming the results of the current study showing admixture within Iranian and Turkish landraces (Strelchenko et al., 2005). Chen et al. (2019) reported that the landraces from Western Asia (Turkey, Syria, Iran, and Iraq) were clustered together and also showed a degree of admixture within the two major clusters identified which separated the landraces from this region from the rest of the landraces and cultivars of other regions. A study of 1,068 wheat landraces from East Asia and West Asia divided the panel into three main subpopulations, interestingly Syrian and Turkish landraces were clustered together, whereas the Iranian landraces showed more genetic similarity with the Afghan landraces than the Syrian and Turkish ones (Lee et al., 2018). The Fertile Crescent which includes modern-day Turkey and Syria is considered the center of origin of the wheat crop, which explains the complex background and admixture present among the landraces collected from these countries (Karagöz, 2014; Baloch et al., 2017). The genetic structure of the current population divided the panel into three major clusters based mainly on their geographic origins with admixture revealing high genetic differentiation between the geographic origin, and the results were similar to previous studies (Morgounov et al., 2016; Baloch et al., 2017; Wang et al., 2017; Rufo et al., 2019). The presence of admixture may be attributed to the historical seed exchange among the regions due to close geographical proximity (Lopes et al., 2015; Morgounov et al., 2016; Alemu et al., 2020).

The fixation index (Fst) is used to measure the genetic differentiation among the populations (Tehseen et al., 2021a). An Fst value of 0.15 and more predicts the presence of a significant genetic differentiation in the subpopulations (Frankham et al., 2002). As a result of high genetic differentiation between the subpopulations, lower levels of gene flow between the subpopulations were expected. The low levels of gene flow could be due to the cultivation of newly developed cultivars in all the countries and less use of traditional bread wheat landraces in the breeding programs. Rufo et al. (2019) also reported low levels of gene flow among the wheat landrace population of Mediterranean origin. Significant differentiation in the two subpopulations was further validated with the analysis of molecular variance (AMOVA), where the majority of the variation (97.4%) was from within the subpopulations. A similar trend was observed when the population stratification was estimated by the DAPC, and when the geographic origin of the landraces was used as a proxy for clustering the populations, most of the genetic variation was observed within the three (95.8%) and eight subpopulations (95.5%), respectively. Whether the genetic variation within the subpopulations is due to the variation that occurred during different domestication events or as a result of introduction from other regions by farmers and traders is still unknown. Many previous studies have reported similar results where most of the variation was accounted for within the subpopulations when compared with between the populations in different hexaploid wheat populations (Zhang et al., 2011; Arora et al., 2014; Joukhadar et al., 2017; Eltaher et al., 2018; Bhatta et al., 2019; Bhattacharjee et al., 2020). Therefore, the selection of parental genotypes from within the subpopulation could be more useful compared with a selection from between the subpopulations. However, this can be changed depending on the breeding objectives. The DAPC analysis divided the landraces into three subpopulations in which most of the landraces of Syrian, Turkish, and Iranian origin were grouped in their respective clusters, and this grouping was in accordance with the geographic proximities of the landraces. The landraces from Iraq and Jordan were genetically closer to the Syrian group, whereas the landraces from Spain, Greece, and Palestine were grouped with Turkish landraces. The countries from these regions have previously been reported to show similar clustering (Kilian et al., 2010; Baloch et al., 2017; Rufo et al., 2019).

Based on the genetic diversity indices when the population stratification was estimated by the STRUCTURE program, subpopulation 2 showed higher genetic diversity than subpopulation 1. Subpopulation 2 consisted of 238 landraces and was mainly composed of landraces from Syria and Turkey, in addition to some landraces from Iran, Greece, and Iraq, whereas subpopulation 1 contained 362 landraces. The presence of higher genetic diversity in subpopulation 2 indicated the potential of this group to be used in breeding programs. In the case of three subpopulations, as estimated by DAPC and PCA, subpopulation 2 was the most diverse as it showed the highest values for genetic diversity indices and was composed of landraces from Syria and Iran. The higher genetic diversity in the Syrian and Iranian landraces has also been previously reported (Zhang et al., 2011; Alipour et al., 2017; Zarei Abbasabad et al., 2017). It is to be noted that there was substantial overlapping of Turkish landraces in both the subpopulations showing their importance which can be utilized for potential economical traits in bread wheat. Several previous reports have reported higher genetic diversity in different panels of Turkish landraces, and it was also reported that in the case of larger population panels, Turkish and Syrian accessions have tended to be genetically closer to each other (Baloch et al., 2017; Yang et al., 2020). When the landraces were divided into different clusters with geographic origin as a proxy, then the highest genetic diversity was observed in Syrian landraces followed by Turkish and Iranian. The results were as expected because 97% of the total landraces belonged to these three geographical regions. There was no significant difference in genetic diversity between the landraces from these three countries, however, it is to be noted that only 8% of the total landraces were from Iran when compared with 62 and 26% from Syria and Turkey, respectively, identifying high genetic diversity in Iranian landraces and their potential use for the exploitation of economically essential traits in breeding programs. The importance of the landraces from these geographic regions has been previously reported as well, which supports the results of the current study (Alipour et al., 2017). Previously, various studies have also reported the genetic diversity among the wheat cultivars from the Mediterranean regions (Nazco et al., 2012; Amallah et al., 2015; Soriano et al., 2016; Rufo et al., 2019). It was observed that almost in all cases, the genetic diversity among the landraces was higher in the landraces than in the cultivars in the region. This could be due to the presence of high genetic variability and their documented durability against biotic and abiotic stresses (Pecetti et al., 1994). The population stratification between the landraces and cultivars has also grouped them both separately because of selected cycles of breeding and allele accumulation in the cultivars (Soriano et al., 2016). The local landraces with high genetic diversity are potential sources of new alleles for the improvement of biotic and abiotic stress resistance when introgressive in the modern cultivars (Nazco et al., 2012).

From these results, we can report that the 600 bread wheat landraces used in the current study, in particular, subpopulation 2, estimated *via* STRUCTURE and DAPC methods, potentially provide broad and important genetic diversity. This diversity could be used in current and future wheat genetic enhancement and breeding research programs around the world. High genetic diversity is an important factor in conducting association mapping studies (GWAS) and marker-assisted selection for the mapping and identification of economically important traits in wheat. In addition, these landraces were collected from eight different countries with diverse agroclimatic conditions, therefore these landraces should also be a useful source of genes to be used in breeding programs addressing the challenges of changing global climate.

## Conclusion

The study provided a detailed population structure and genetic diversity analysis of 600 bread wheat landraces collected from eight countries preserved at the ICARDA genebank. Clustering analysis showed distinct population structures in the landraces. The landraces were mainly divided into Turkish, Syrian, and Iranian groups with significant overlapping. This admixture is a result of historical seed exchange between these countries through farmers and traders due to their close geographical proximity to each other. The genetic diversity indices represented high genetic diversity in these wheat landraces. These landraces were collected from a wide range of agroclimatic zones, as a result possess high diversity and capacity to tolerate and resist various abiotic and biotic constraints, and could hence be used as a potential source of new genes/alleles for the genetic enhancement of hexaploid wheat. Therefore, sustainable conservation and use of these landraces preserved in the genebank is important for future breeding strategies of wheat breeding programs worldwide.

## Data Availability

The data sets presented in this study can be found in online repositories. The names of the repository/repositories and accession number(s) can be found in the article/Supplementary Material.
