# Supplementary figures and images for: Exploring the Genetic Diversity and Population Structure of Wheat Landrace Population Conserved at ICARDA Genebank
Source: Front Genet. 2022 Jun 15;13:900572. doi: 10.3389/fgene.2022.900572 (PMC9240388; doi:10.3389/fgene.2022.900572)

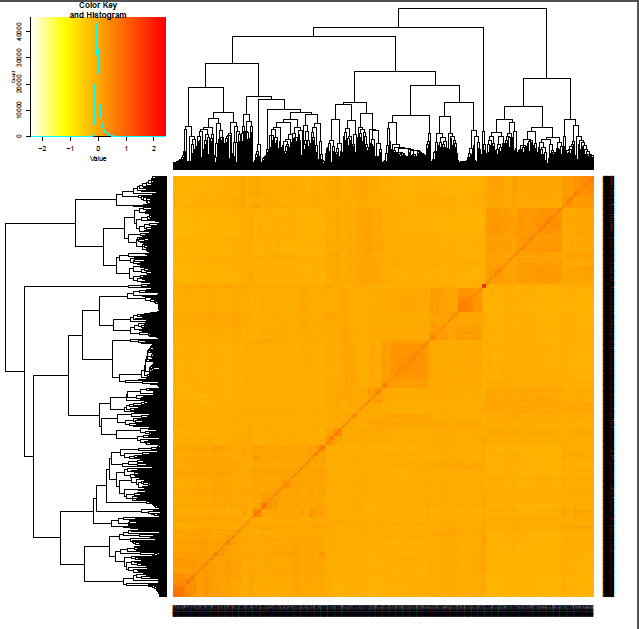

Supplement: Supplementary file 3 [file Image1.PNG]
